# Proteomic aging atlas identifies senoproteins as drivers of vascular and systemic senescence

**DOI:** 10.1093/lifemedi/lnag003

**Published:** 2026-01-29

**Authors:** Chongwei Bi, Mo Li

**Affiliations:** Bioinformatics Laboratory, The First Hospital of Jilin University, Changchun 130012, China; Bioscience Program, Biological and Environmental Science and Engineering Division (BESE), King Abdullah University of Science and Technology (KAUST), Thuwal 23955, Saudi Arabia; KAUST Center of Excellence for Smart Health (KCSH), Thuwal 23955, Saudi Arabia

Mammalian aging is a natural process marked by the progressive decline of organ function and increased vulnerability to chronic diseases. Organs follow distinct aging trajectories—some age earlier than others—yet, they act in concert to shape the overall organismal aging [1]. However, our understanding of organ-specific aging patterns and the heterogeneity of systemic aging remains limited, and the mechanisms underlying multi-organ interaction during aging are still unclear. These gaps hindered the development of reliable aging biomarkers and quantitative metrics to accurately assess organ-specific biological age and to effectively evaluate interventions targeting specific aging processes.

Proteins are fundamental to cellular function, and loss of proteostasis is recognized as a major hallmark of aging [2]. However, proteomic alterations in aged human organs remain poorly characterized, as most studies focused primarily on plasma proteomics while overlooking the proteostasis decline in organ-specific context. A recent study by Ding et al. closed these long-standing gaps in aging research by conducting comprehensive proteomic, transcriptomic, and histological analyses across five decades of life in 13 kinds of human tissues [3]. This large-scale dataset reveals widespread transcriptome–proteome decoupling and an unsynchronized progressive decline in proteostasis across multiple organs during aging. The authors further developed proteomic age clocks to characterize heterogeneous dynamics in tissue-specific aging trajectories and identified candidate senoproteins driving vascular and systemic aging.

**Figure 1.**
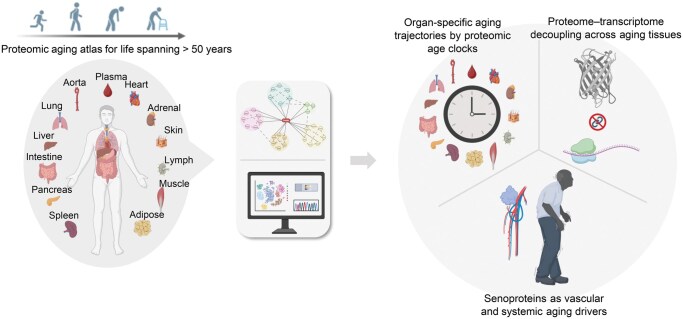
**The proteomic aging atlas constructed from 13 tissues spanning over 50 years of life.** Comprehensive proteomic and transcriptomic profiling revealed age-associated divergence between the proteome and transcriptome, enabled the construction of tissue-specific proteomic age clocks to capture diverse aging trajectories of organs, and uncovered candidate senoproteins as potential aging drivers contributing to vascular and multi-organ degeneration. Created in BioRender. Bi. C (2025)

Utilizing state-of-the-art mass spectrometry and parallel transcriptomic assay, the authors conducted a quantitative proteomic analysis of tissues from individuals aged 14 to 68, encompassing seven bodily systems: cardiovascular (heart and aorta), digestive (liver, pancreas, and intestine), immune (spleen and lymph node), endocrine (adrenal gland and white adipose), respiratory (lung), integumentary (skin), and musculoskeletal (muscle), along with blood samples (Fig. 1). A total of 12,771 distinct proteins were identified, covering the protein-coding genes across all chromosomes. Notably, when comparing the proteomic profiles with transcriptomic data, they observed only moderate correlation between protein and mRNA levels across multiple organs. This positive correlation declined markedly with age, indicating an age-dependent decoupling of the proteome and transcriptome. Further analysis provided insights that the translational levels of several key proteins involved in protein synthesis, as well as proteins responsible for folding, assembly, and transport, were found to decrease across multiple tissues with age. Alongside the deterioration of protein quality control, organ aging was also marked by excessive activation of immune pathways, as evidenced by the accumulation of amyloid proteins, immunoglobulins, and complement components.

The comprehensive age-stratified proteomic dataset enabled the identification of age-dependent differentially expressed proteins (DEPs) that exhibited altered expression in at least one organ during human aging. The majority of these DEPs were found to be tissue-specific, with only approximately 40% shared across at least two tissues, highlighting the organ-selective deterioration of tissue function with age. Nevertheless, the authors identified a subset of upregulated DEPs that consistently increased with age across multiple tissues. These proteins were predominantly involved in complement activation, immune response, and amyloid fibril formation. In contrast, commonly downregulated DEPs across tissues were enriched in proteins associated with Golgi vesicle transport and protein maturation. Notably, 31 proteins were uniformly downregulated in more than 6 tissues without upregulation in any organ. These were designated as ubiquitous downregulated proteins with aging, and include PRPF4, a key component involved in pre-mRNA splicing. Conversely, 29 proteins were consistently upregulated across more than 6 tissues and termed ubiquitous upregulated proteins with aging (UUPA). These proteins were enriched in pathways related to complement activation, inflammatory responses, and cell adhesion. Among them, serum amyloid P-component (SAP, encoded by APCS)—a canonical amyloid-related factor and a representative example of mRNA-protein decoupling, particularly evident in the liver—emerged as the most prominent UUPA.

Further efforts were made to establish an aging hallmark assessment system to elucidate proteomic alterations in aging organs, using aging hallmarks such as cell cycle arrest, genomic instability, loss of heterochromatin, inflammation, and fibrosis. Among these hallmarks, mitochondrial proteins involved in mitochondrial biogenesis, homeostasis, and respiration were found to be consistently downregulated across multiple organs, as revealed by gene set enrichment analysis, highlighting the important role of mitochondrial dysfunction in aging. Epigenetic instability was also evident, with YTHDC1 identified as the most consistently downregulated epigenetic regulator across tissues. Recognizing that proteins often function as complexes, the authors analysed 816 protein complexes, integrating both proteomic and transcriptomic data. Upregulated complexes were predominantly enriched in inflammasome-related pathways, while downregulated complexes were associated with nuclear hormone receptors, nuclear transport machinery, and spliceosomes. Finally, the study endorsed the connection between tissue aging and increased vulnerability to chronic diseases, as demonstrated by the age-associated increase in expression levels of 48 disease-related risk factor proteins.

To address the heterogeneity of aging across human organs, the authors developed proteomic age clocks for the 13 surveyed tissues. These clocks served as multi-time-point protein-based navigators to track tissue-specific transition points during the aging process. Using a sliding window-based cumulative analysis, they discovered that many tissues underwent substantial proteomic remodeling around age 50 years. Notably, the aorta, spleen, and adrenal gland exhibited significant protein expression changes as early as age 30 years, with the aorta showing the most pronounced and continuous proteomic fluctuations throughout the lifespan. Early proteomic alterations at age 30 years were primarily linked to ribosome function and translation, whereas later transitions were enriched in pathways related to RNA splicing, protein targeting, and subcellular localization. These findings demonstrated the complexity and variability of aging across organs, with the aorta identified as an early and prominently affected organ.

Secretory proteins are essential for organ function and mediate inter-organ communication. By analysing 1140 secretory proteins from each organ, the authors identified 579 distinct senescence-associated secretory phenotype proteins. Among these, CXCL12—a chemokine—was notably accumulated in nine tissues and showed age-associated upregulation. Inter-organ communication networks were assessed using CellPhoneDB, revealing that the aorta, immune tissues, and endocrine organs exhibited extensive interactions with other tissues, which intensified with age. In plasma-mediated interactions, GAS6, a ligand whose expression increased in both plasma and its tissue of origin (aorta), showed enhanced potential to interact with TAM receptors that are broadly expressed across various tissues. Functional experiments demonstrated that treatment of human aortic endothelial cells (hAECs) and human vascular smooth muscle cells with recombinant human GAS6 protein accelerated cellular senescence and increased inflammatory cytokine levels. *In vivo*, GAS6-treated mice exhibited declined physical performance and accelerated senescence phenotypes in vascular and multiple other target organs.

The development of blood-based biomarkers is crucial for enabling non-invasive evaluation of organ aging. By comparing plasma and organ proteomic data, the authors identified 211 plasma–tissue paired DEPs that exhibited synchronized age-related changes. Most of these paired DEPs were uniquely associated with a single tissue, while seven were upregulated across multiple tissues and showed significant accumulation in aging plasma. Among them was GPNMB, a previously reported senescence-associated antigen known to increase with age in plasma, as confirmed by several studies. Leveraging these plasma–tissue paired DEPs, the authors constructed plasma protein-based age clocks as an alternative to tissue protein-based clocks for assessing organ age. This plasma-derived clock demonstrated comparable age-prediction accuracy to that of the tissue protein matrix-derived clock.

Given that the vasculature was identified as one of the earliest and most affected systems during aging, Ding et al. investigated whether specific vascular wall interaction proteins accumulating in plasma could contribute to vascular aging. Treatment of hAECs with recombinant forms of selected plasma proteins revealed that seven senoproteins—GPNMB, COMP, HTRA1, SLPI, IGFBP7, NEGR1, and NOTCH3—were capable of inducing vascular endothelial senescence. Transcriptomic analysis further demonstrated that exposure to these proteins activated canonical senescence-associated genes and upregulated pathways involved in cellular senescence, oxidative stress, and inflammation. Additional experiments showed that GPNMB, HTRA1, and IGFBP7 could also induce senescence phenotypes in cell types beyond the endothelium, and mice treated with GPNMB exhibited typical aging phenotypes. These findings suggest that senoproteins secreted by aging tissues can amplify senescence-promoting signals, thereby mediating inter-organ aging communication and driving both vascular and multi-organ degeneration. Therefore, the authors propose naming these senescence-promoting secretory proteins expressed by aging tissues “senokines.”

Together, this work presents the first multi-organ, high-resolution proteomic atlas of human aging, uncovering the complexity and heterogeneity of organ aging from a proteomic perspective. The constructed protein age clocks not only delineate the dynamic aging trajectories of human organs but also hold promise as quantitative tools for evaluating interventions targeting specific aspects of the aging process [4^, ^5]. By identifying a series of senescence-associated proteins—including potential aging drivers of senoproteins—the study offers new opportunities for protein-targeted geroprotective strategies. Collectively, these findings lay a critical foundation for future efforts to decode, monitor, and potentially reverse human aging.
